# Crystal structure of (*Z*)-1-(ferrocenylethyn­yl)-10-(phenyl­imino)­anthracen-9(10*H*)-one from synchrotron X-ray powder diffraction

**DOI:** 10.1107/S1600536814025252

**Published:** 2014-11-26

**Authors:** Eiji Nishibori, Shinobu Aoyagi, Makoto Sakata, Ryota Sakamoto, Hiroshi Nishihara

**Affiliations:** a Division of Physics, Faculty of Pure and Applied Sciences, Center for Integrated Research in Fundamental Science and Engineering, Tsukuba Research Center for Interdisciplinary Materials Science, University of Tsukuba, 1-1-1 Tennodai, Tsukuba, Ibaraki 305-8571, Japan; b Department of Information and Biological Sciences, Nagoya City University, Nagoya 467-8501, Japan; c Japan Synchrotron Radiation Research Institute, SPring-8, 1-1-1, Kouto, Sayo-cho, Sayo-gun, Hyogo 679-5198, Japan; d Department of Chemistry, The University of Tokyo, 7-3-1, Hongo, Bunkyo-ku, Tokyo 113-0033, Japan

**Keywords:** structure determination, powder diffraction, synchrotron radiation, *D–A* conjugated complex, ferrocen­yl–anthracen-9(10*H*)-one, π–π inter­actions, C—H⋯π inter­actions

## Abstract

In the title compound, [Fe(C_5_H_5_)(C_27_H_16_NO)], designed and synthesized to explore a new electron-donor (*D*) and -acceptor (*A*) conjugated complex, the two cyclo­penta­dienyl rings adopt an eclipsed conformation. The anthracene tricycle is distorted towards a butterfly conformation and the mean planes of the outer benzene rings are inclined each to other at 22.7 (3)°. In the crystal, mol­ecules are paired into inversion dimers *via* π–π inter­actions. Weak inter­molecular C—H⋯π inter­actions link further these dimers into one-dimensional columns along the *b* axis, with the ferrocenylethynyl arms arranged between the stacks to fill the voids.

## Abstract 2

In a ferrocen­yl–anthracen-9(10*H*)-one compound that has been designed and synthesized to explore a new electron-donor (*D*) and -acceptor (*A*) conjugated complex, the two cyclo­penta­dienyl rings adopt an eclipsed conformation. The anthracene tricycle is distorted towards a butterfly conformation.

## Chemical context   

Compounds containing a mixture of electron-donor (*D*) and -acceptor (*A*) mol­ecules have attracted much attention owing to their unique structures and various characteristic properties (Alberola *et al.*, 2003 ▶; Ferraris *et al.*, 1973 ▶). *D–A*-conjugated complexes of ferrocenylethynylanthra­quinones (FcAq) demonstrate guest-mol­ecule absorption and valence tautomerization *etc*. We have synthesized the title compound 1-(ferrocenylethyn­yl)-10-(phenyl­imino)­anthracen-9(10*H*)-one [1-(Fc)AqPHI] and herein we report its crystal structure, determined by synchrotron radiation (SR) X-ray powder diffraction.
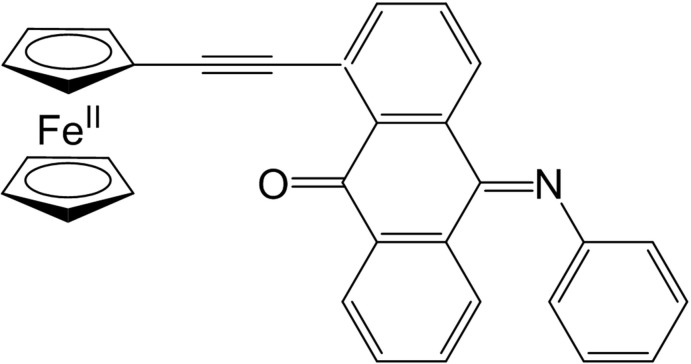


## Structural commentary   

**Figure 1:**
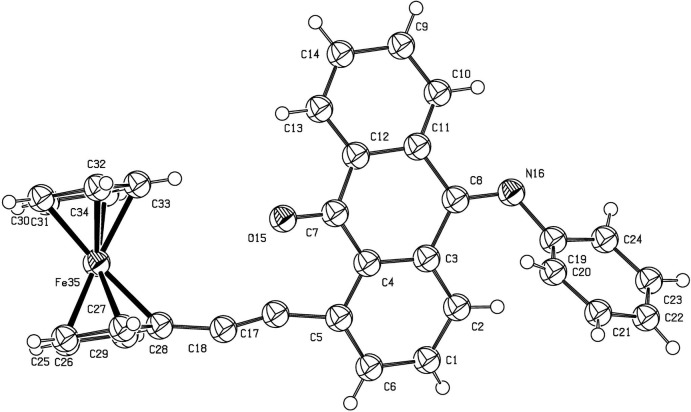
The mol­ecular structure of 1-FcAqPHI, showing the atomic numbering and 50% probability displacement spheres.

Fig. 1 ▶ shows the mol­ecular structure of 1-(Fc)AqPHI, which contains two five-membered and four six-membered carbon rings. The two cyclo­penta­dienyl rings adopt an eclipsed conformation. The anthracene tricycle is distorted towards a butterfly conformation, and the mean planes of the outer benzene rings are inclined each to other at 22.7 (3)°.

## Supra­molecular features   

**Figure 2:**
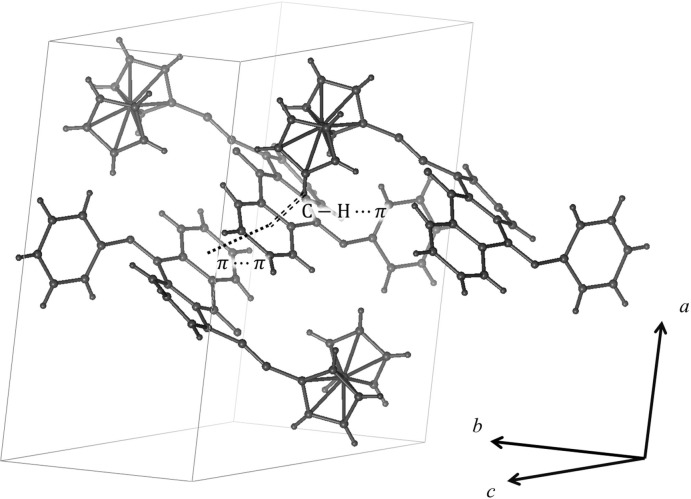
The crystal packing of 1-FcAqPHI. The π–π and C—H⋯π contacts are shown as dotted and dashed lines, respectively.

In the crystal (Fig. 2 ▶), π–π inter­actions (Table 1 ▶) between the Aq parts of the mol­ecules pair them into inversion dimers, and weak inter­molecular C—H⋯π inter­actions (Table 2 ▶) link further these dimers into one-dimensional columns along the *b* axis, with the ferrocenylethynyl arms arranged between the stacks to fill the voids.

## Database survey   

In the reported examples compiled in the Cambridge Structural Database (Groom & Allen, 2014 ▶) of Fc-Aq compounds, 1,4-Fc_2_Aq (Kondo *et al.*, 2006 ▶), 1,5-Fc_2_Aq (Murata *et al.*, 2001 ▶) and 1,4-(FcPh)_2_Aq (Sachiko *et al.*, 2013 ▶), the cyclo­penta­dienyl (CP) rings have an eclipsed conformation except for only in one low-temperature phase of 1,4-(FcPh)_2_Aq. Similar π–π stacking interactions were observed in the other FcAq compounds, *viz.* 1,4-Fc_2_Aq, 1,5-Fc_2_Aq and 1,4-(FcPh)_2_Aq. Distances between the ring centroids cover the range from 4.09 Å in 1,4 Fc_2_Aq down to 3.68 Å in 1,2-(FcPh)_2_Aq. The smallest perpendicular distance for all the materials was close to 3.45 Å [3.45, 3.43 and 3.42 Å for 1,4-Fc_2_Aq, 1,5-Fc_2_Aq and 1,4-(FcPh)_2_Aq, respectively]. C—H⋯π inter­actions are also found in 1,4-Fc_2_Aq, 1,5-Fc_2_Aq and 1,2-(FcPh)_2_Aq. Two kinds of C—H⋯π inter­actions in 1,4-Fc_2_Aq connect the CP rings and the rings of the Aq groups of neighbouring mol­ecules. A C—H⋯π inter­action in 1,5-Fc_2_Aq links a CH– group from the Aq unit and a CP ring of Fc fragment. There are three C—H⋯π inter­actions in 1,2-(FcPh)_2_Aq.

## Synthesis and crystallization   

Under a nitro­gen atmosphere, 1-bromo-10-(phenyl­imino)­anthracen-9(10*H*)-one (89 mg, 0.24 mmol), ethynylferrocene (47 mg, 0.22 mmol), Pd(PPh_3_)2Cl_2_ (3.1 mg), and CuI (5 mg) were suspended in Et_3_N (15 ml). After refluxing for 5 h, Et_3_N was removed *in vacuo*, and the resultant residue was dissolved in CH_2_Cl_2_. The solution was washed with water (150 ml), and dried over Na_2_SO_4_. After evaporation of the solvent, the crude product was purified with alumina column chromatography (activity II–III) with a mixture of di­chloro­methane and hexane (1:2 *v*/*v*) as eluent. The third fraction was collected, and produced a red–brown solid of the title compound (yield: 30 mg, 33%). Very small single crystals unsuitable for conventional X-ray structure analysis were obtained by recrystallization from di­chloro­methane–hexane. ^1^H NMR (400 MHz, CDCl_3_): δ 8.1–8.5 (*m*, 2H), 7.0–7.9 (*m*, 8H), 6.80 (*d*, 2H), 4.1–4.8 (*m*, 9H). IR (KBr pellet): 2208 (ν C=C/ cm^−1^), 1668 (ν C=O/ cm^−1^), 1483 (ν C=N/ cm^−1^). MALDI–TOF–MS: *m*/*z* = 490.1.

## Refinement details   

The size of 1-(Fc)AqPHI crystals was small, less than 1 µm. SR powder-diffraction techniques were employed for the structure determination. The powder crystallites were installed in a 0.4 mm glass capillary. The X-ray powder diffraction data were measured using a large Debye–Scherrer camera with an imaging-plate (IP) as a detector installed at SPring-8 BL02B2 (Nishibori *et al.*, 2001 ▶). The CeO_2_ (NIST SRM674*a*) standard powder sample was used for wavelength calibration. The calibrated wavelength was 0.80200 (1) Å. The powder profile was measured at 100 K with 120 min X-ray exposure time.

Indexing was carried out using the program *DICVOL04* (Boultif & Louer, 2004 ▶). The first 21 peaks of the powder pattern were completely indexed on the basis of a monoclinic cell. The figure of merit *F*(21) was 63.2. The space group *P*2_1_/*n* was assigned on the basis of systematic extinctions.

**Figure 3:**
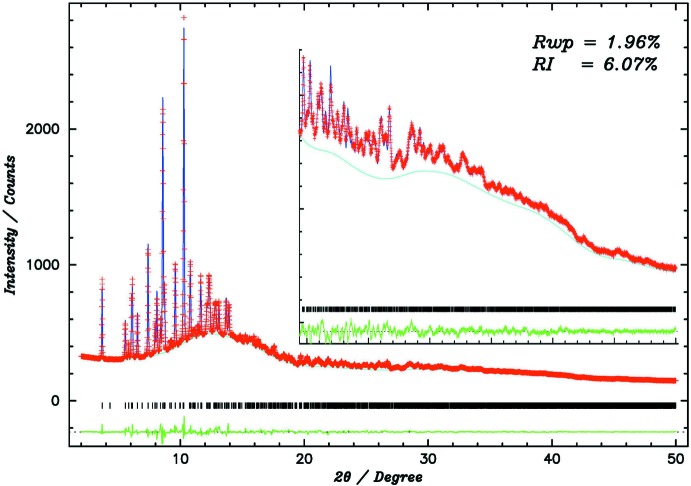
The fitting results of the final Rietveld refinement. The experimental profile is indicated by red crosses. The calculated profile is shown as a solid blue line, and the cyan line indicates the calculated background. The difference profile is shown as the bottom solid green line. The vertical black bars correspond to the calculated positions of the Bragg peaks.

The lattice constants were refined by the Le Bail method using the program *SP* (Nishibori *et al.*, 2007 ▶). The crystal structure was determined from powder diffraction data using a direct-space method with a genetic algorithm (Harris *et al.*, 1998 ▶; Nishibori *et al.*, 2008 ▶). The mol­ecular structure model for GA was constructed using similar structures, 1,4-Fc_2_Aq, 1,5-Fc_2_Aq, and 1,8-Fc_2_Aq (Kondo *et al.*, 2006 ▶, Murata *et al.*, 2001 ▶). The chemically equivalent distances were equal in the model. GA analysis using the *P*2_1_/*n* space group was performed. A solution was obtained. The rigid-body Rietveld refinement was initially carried out using the program *SP*. Restraint Rietveld analysis was employed for the final refinement, with chemically equivalent distances being equal. Displacement parameters were refined as isotropic. Four common *U*_iso_ parameters were refined for several groups of C atoms in the Aq fragment: C1–C14, phenyl ring C19–C24, and CP rings C25–C29 and C30–C34. One common *U*_iso_ parameter was also refined for carbon atoms at the *D*–*A* junction (C17 and C18). *U*_iso_ for H atoms connected to the Aq and Ph parts were fixed at 0.05 Å^2^. *U_iso_* for H atoms connected to the C25–C29 and C30–C34 CP rings were fixed at 0.09 Å^2^ and 0.04 Å^2^, respectively. A split-type pseudo-Voigt profile function (Toraya, 1990 ▶) was used with strain broadening (Stephens, 1999 ▶). Results of the Rietveld refinements are shown in Fig. 3 ▶. Crystal data, data collection and structure refinement details are summarized in Table 3 ▶.

## Supplementary Material

Crystal structure: contains datablock(s) global, I, 1FcAqPHI-100K_powder_data. DOI: 10.1107/S1600536814025252/cv5475sup1.cif

Rietveld powder data: contains datablock(s) I. DOI: 10.1107/S1600536814025252/cv5475Isup2.rtv

Supporting information file. DOI: 10.1107/S1600536814025252/cv5475Isup3.mol

CCDC reference: 1034683

Additional supporting information:  crystallographic information; 3D view; checkCIF report

## Figures and Tables

**Table 1:** interactions () *Cg*1 is the centroid of the C9C14 ring and *Cg*1_Perp is the perpendicular distance from *Cg*1^i^ to the C9C14 ring.

*Cg*1*Cg*1^i^	*Cg*1_Perp^i^
3.802(3)	3.486(2)

Symmetry code: (i) *x*, *y*+1, *z*+2.

**Table 2:** Hydrogen-bond geometry (, ) *Cg*1 is the centroid of the C9C14 ring.

*D*H*A*	*D*H	H*A*	*D* *A*	*D*H*A*
C34H56*Cg*1^i^	0.87(1)	2.86(1)	3.588(4)	143(1)

Symmetry code: (i) 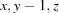.

**Table 3:** Experimental details

Crystal data	
Chemical formula	[Fe(C_5_H_5_)(C_27_H_16_NO)]
*M*_r_	491.35
Crystal system, space group	Monoclinic, *P*2_1_/*n*
Temperature (K)	100
*a*, *b*, *c* ()	15.9542(3), 8.5087(2), 16.7212(4)
()	99.070(2)
*V* (^3^)	2241.51(9)
*Z*	4
Radiation type	Synchrotron, = 0.80200
(mm^1^)	0.96
Specimen shape, size (mm)	Cylinder, 3.0 0.4

Data collection	
Diffractometer	Large DebyeScherrer camera
Specimen mounting	Capillary
Data collection mode	Transmission
Scan method	Stationary detector
2 values ()	2_fixed_ = 0.0178.68

Refinement	
*R* factors and goodness of fit	*R*_p_ = 0.010, *R*_wp_ = 0.020, *R*_exp_ = 0.01, *R*_Bragg_ = 0.061, *R*(*F*) = 0.040, *R*(*F* ^2^) = 0.061, ^2^ = 6.554
No. of data points	7868
No. of parameters	180
No. of restraints	241
H-atom treatment	All H-atom parameters refined

Computer programs: local software (Nishibori *et al.*, 2001 ▶), *SP* (Nishibori *et al.*, 2007 ▶), *GAIA* (Nishibori *et al.*, 2008 ▶), *pyMOL* (DeLano, 2002 ▶) and *publCIF* (Westrip, 2010 ▶).

## supplementary crystallographic information

### Crystal data

**Table:**

[Fe(C_5_H_5_)(C_27_H_16_NO)]	*Z* = 4
*M**_r_* = 491.35	*F*(000) = 1016.0
Monoclinic, *P*2_1_/*n*	*D*_x_ = 1.456 Mg m^−^^3^
Hall symbol: -P 2yn	Synchrotron radiation, λ = 0.80200 Å
*a* = 15.9542 (3) Å	µ = 0.96 mm^−^^1^
*b* = 8.5087 (2) Å	*T* = 100 K
*c* = 16.7212 (4) Å	orange
β = 99.070 (2)°	cylinder, 3 × 0.4 mm
*V* = 2241.51 (9) Å^3^	

### Data collection

**Table:**

Large Debye-Scherrer camera diffractometer	Data collection mode: transmission
Radiation source: synchrotron, SPring-8 BL02B2	Scan method: Stationary detector
Si(111) monochromator	2θ_fixed_ = 0.01-78.68
Specimen mounting: capillary	

### Refinement

**Table:**

Refinement on *I*_net_	Excluded region(s): none
Least-squares matrix: full	Profile function: split-Pseudo-Voigt
*R*_p_ = 0.010	180 parameters
*R*_wp_ = 0.020	241 restraints
*R*_exp_ = 0.01	0 constraints
*R*_Bragg_ = 0.061	All H-atom parameters refined
*R*(*F*) = 0.040	Weighting scheme based on measured s.u.'s
*R*(*F*^2^) = 0.061	(Δ/σ)_max_ = 0.02
χ^2^ = 6.554	Background function: split-Pearson7 and polynomial function
7868 data points	Preferred orientation correction: none

### Fractional atomic coordinates and isotropic or equivalent isotropic displacement parameters (Å^2^)

**Table:**

	*x*	*y*	*z*	*U*_iso_*/*U*_eq_	
C1	0.1031 (3)	−0.0140 (6)	0.7090 (2)	0.062 (6)*	
C2	0.0713 (4)	0.1155 (7)	0.7483 (2)	0.062 (6)*	
C3	0.0924 (2)	0.1347 (4)	0.8333 (2)	0.062 (6)*	
C4	0.1447 (2)	0.0262 (4)	0.8777 (2)	0.062 (6)*	
C5	0.1856 (2)	−0.0903 (4)	0.8380 (2)	0.062 (6)*	
C6	0.1675 (4)	−0.1075 (6)	0.7529 (3)	0.062 (6)*	
C7	0.1611 (2)	0.0357 (5)	0.9640 (2)	0.062 (6)*	
C8	0.0473 (2)	0.2547 (3)	0.8772 (2)	0.062 (6)*	
C9	−0.0113 (4)	0.2770 (7)	1.0844 (2)	0.062 (6)*	
C10	−0.0154 (3)	0.2981 (7)	1.0026 (3)	0.062 (6)*	
C11	0.0441 (3)	0.2287 (4)	0.9646 (2)	0.062 (6)*	
C12	0.0980 (2)	0.1093 (5)	1.0053 (2)	0.062 (6)*	
C13	0.1014 (4)	0.0866 (7)	1.0862 (2)	0.062 (6)*	
C14	0.0459 (5)	0.165 (1)	1.1260 (2)	0.062 (6)*	
O15	0.2135 (3)	−0.0534 (6)	1.0027 (2)	0.125 (6)*	
N16	−0.0023 (3)	0.3603 (5)	0.8385 (3)	0.009 (5)*	
C17	0.2217 (3)	−0.2172 (6)	0.8904 (3)	0.20 (1)*	
C18	0.2734 (2)	−0.3221 (5)	0.9182 (2)	0.23 (1)*	
C19	0.0064 (2)	0.4110 (3)	0.7568 (2)	0.025 (6)*	
C20	0.0868 (2)	0.4571 (8)	0.7408 (3)	0.025 (6)*	
C21	0.0943 (3)	0.5259 (9)	0.6656 (4)	0.025 (6)*	
C22	0.0209 (4)	0.562 (1)	0.6101 (3)	0.025 (6)*	
C23	−0.0599 (3)	0.530 (1)	0.6298 (3)	0.025 (6)*	
C24	−0.0669 (2)	0.4640 (8)	0.7053 (3)	0.025 (6)*	
C25	0.3354 (2)	−0.7069 (3)	1.0118 (2)	0.062 (8)*	
C26	0.4075 (1)	−0.6142 (3)	1.0390 (2)	0.062 (8)*	
C27	0.3895 (1)	−0.4585 (4)	1.0129 (2)	0.062 (8)*	
C28	0.3061 (1)	−0.4548 (4)	0.9695 (2)	0.062 (8)*	
C29	0.2727 (2)	−0.6084 (4)	0.9689 (1)	0.062 (8)*	
C30	0.2582 (2)	−0.6324 (4)	1.1771 (2)	0.060 (9)*	
C31	0.3326 (2)	−0.5478 (5)	1.2063 (1)	0.060 (9)*	
C32	0.3193 (2)	−0.3895 (4)	1.1825 (2)	0.060 (9)*	
C33	0.2366 (2)	−0.3765 (4)	1.1386 (2)	0.060 (9)*	
C34	0.1988 (1)	−0.5266 (4)	1.1354 (2)	0.060 (9)*	
FE35	0.3053 (1)	−0.5312 (2)	1.0845 (1)	0.0324 (5)*	
H36	0.0822 (5)	−0.0379 (8)	0.6558 (2)	0.05*	
H37	0.0391 (6)	0.1912 (9)	0.7173 (2)	0.05*	
H38	0.1931 (8)	−0.186 (1)	0.7267 (3)	0.05*	
H39	−0.045 (1)	0.339 (2)	1.1130 (5)	0.05*	
H40	−0.0511 (6)	0.375 (1)	0.9759 (4)	0.05*	
H41	0.1469 (7)	0.032 (2)	1.1154 (3)	0.05*	
H42	0.045 (3)	0.144 (5)	1.180 (1)	0.05*	
H43	0.1333 (3)	0.456 (2)	0.7821 (4)	0.05*	
H44	0.1475 (3)	0.554 (2)	0.6542 (7)	0.05*	
H45	0.0260 (5)	0.596 (3)	0.5582 (5)	0.05*	
H46	−0.1083 (4)	0.563 (3)	0.5956 (6)	0.05*	
H47	−0.1190 (2)	0.463 (2)	0.7233 (5)	0.05*	
H48	0.3304 (3)	−0.8071 (3)	1.0202 (3)	0.09*	
H49	0.4548 (2)	−0.6471 (4)	1.0672 (2)	0.09*	
H50	0.4237 (1)	−0.3786 (4)	1.0220 (3)	0.09*	
H51	0.2223 (2)	−0.6373 (5)	0.9461 (2)	0.09*	
H52	0.2505 (2)	−0.7326 (4)	1.1834 (2)	0.04*	
H53	0.3788 (2)	−0.5865 (6)	1.2337 (1)	0.04*	
H54	0.3555 (3)	−0.3131 (4)	1.1935 (2)	0.04*	
H55	0.2131 (2)	−0.2910 (4)	1.1170 (2)	0.04*	
H56	0.1479 (1)	−0.5501 (5)	1.1113 (2)	0.04*	

### Geometric parameters (Å, º)

**Table:**

Fe35—C25	2.032 (3)	C21—C22	1.409 (8)
Fe35—C26	2.032 (3)	C22—C23	1.407 (8)
Fe35—C27	2.032 (3)	C23—C24	1.402 (8)
Fe35—C28	2.032 (4)	C25—C26	1.410 (4)
Fe35—C29	2.030 (3)	C25—C29	1.411 (4)
Fe35—C30	2.018 (4)	C26—C27	1.410 (4)
Fe35—C31	2.019 (2)	C27—C28	1.412 (3)
Fe35—C32	2.018 (4)	C28—C29	1.411 (5)
Fe35—C33	2.016 (4)	C30—C31	1.408 (5)
Fe35—C34	2.017 (3)	C30—C34	1.409 (4)
O15—C7	1.234 (6)	C31—C32	1.411 (5)
N16—C8	1.300 (5)	C32—C33	1.410 (5)
N16—C19	1.460 (6)	C33—C34	1.410 (5)
C1—C2	1.417 (7)	C1—H36	0.922 (5)
C1—C6	1.411 (7)	C2—H37	0.929 (9)
C2—C3	1.418 (5)	C6—H38	0.928 (11)
C3—C4	1.380 (5)	C9—H39	0.937 (16)
C3—C8	1.505 (4)	C10—H40	0.934 (10)
C4—C5	1.409 (5)	C13—H41	0.932 (13)
C4—C7	1.428 (5)	C14—H42	0.923 (19)
C5—C6	1.414 (6)	C20—H43	0.931 (7)
C5—C17	1.451 (6)	C21—H44	0.930 (9)
C7—C12	1.450 (5)	C22—H45	0.930 (13)
C8—C11	1.487 (5)	C23—H46	0.929 (12)
C9—C10	1.371 (6)	C24—H47	0.928 (5)
C9—C14	1.423 (9)	C25—H48	0.870 (4)
C10—C11	1.358 (7)	C26—H49	0.871 (4)
C11—C12	1.432 (5)	C27—H50	0.870 (4)
C12—C13	1.359 (5)	C29—H51	0.869 (4)
C13—C14	1.363 (9)	C30—H52	0.870 (5)
C17—C18	1.254 (6)	C31—H53	0.869 (4)
C18—C28	1.464 (5)	C32—H54	0.870 (5)
C19—C20	1.407 (5)	C33—H55	0.870 (5)
C19—C24	1.413 (5)	C34—H56	0.871 (3)
C20—C21	1.409 (9)		
			
C25—Fe35—C26	40.60 (12)	Fe35—C25—C26	69.70 (16)
C25—Fe35—C27	68.34 (14)	Fe35—C25—C29	69.61 (16)
C25—Fe35—C28	68.40 (14)	C26—C25—C29	108.0 (2)
C25—Fe35—C29	40.66 (13)	Fe35—C26—C25	69.70 (15)
C25—Fe35—C30	107.27 (15)	Fe35—C26—C27	69.70 (13)
C25—Fe35—C31	121.22 (17)	C25—C26—C27	108.1 (2)
C25—Fe35—C32	156.79 (16)	Fe35—C27—C26	69.69 (16)
C25—Fe35—C33	160.98 (16)	Fe35—C27—C28	69.67 (15)
C25—Fe35—C34	124.13 (15)	C26—C27—C28	108.0 (3)
C26—Fe35—C27	40.61 (12)	Fe35—C28—C18	138.3 (2)
C26—Fe35—C28	68.39 (12)	Fe35—C28—C27	69.66 (19)
C26—Fe35—C29	68.36 (13)	Fe35—C28—C29	69.61 (16)
C26—Fe35—C30	123.85 (15)	C18—C28—C27	122.6 (3)
C26—Fe35—C31	107.17 (15)	C18—C28—C29	127.7 (3)
C26—Fe35—C32	121.27 (15)	C27—C28—C29	107.9 (3)
C26—Fe35—C33	156.95 (15)	Fe35—C29—C25	69.73 (15)
C26—Fe35—C34	160.72 (15)	Fe35—C29—C28	69.74 (18)
C27—Fe35—C28	40.67 (10)	C25—C29—C28	108.1 (2)
C27—Fe35—C29	68.37 (14)	Fe35—C30—C31	69.66 (17)
C27—Fe35—C30	160.57 (15)	Fe35—C30—C34	69.54 (18)
C27—Fe35—C31	123.92 (15)	C31—C30—C34	108.0 (3)
C27—Fe35—C32	107.32 (15)	Fe35—C31—C30	69.52 (16)
C27—Fe35—C33	121.49 (16)	Fe35—C31—C32	69.50 (17)
C27—Fe35—C34	157.21 (16)	C30—C31—C32	108.0 (3)
C28—Fe35—C29	40.65 (13)	Fe35—C32—C31	69.59 (17)
C28—Fe35—C30	157.16 (14)	Fe35—C32—C33	69.5 (2)
C28—Fe35—C31	160.80 (16)	C31—C32—C33	108.0 (3)
C28—Fe35—C32	124.12 (16)	Fe35—C33—C32	69.6 (2)
C28—Fe35—C33	107.50 (15)	Fe35—C33—C34	69.58 (17)
C28—Fe35—C34	121.64 (13)	C32—C33—C34	108.0 (3)
C29—Fe35—C30	121.48 (15)	Fe35—C34—C30	69.57 (16)
C29—Fe35—C31	156.90 (18)	Fe35—C34—C33	69.50 (17)
C29—Fe35—C32	160.96 (16)	C30—C34—C33	108.0 (2)
C29—Fe35—C33	124.24 (15)	C2—C1—H36	121.2 (6)
C29—Fe35—C34	107.56 (15)	C6—C1—H36	120.0 (7)
C30—Fe35—C31	40.83 (14)	C1—C2—H37	119.2 (5)
C30—Fe35—C32	68.83 (14)	C3—C2—H37	120.2 (6)
C30—Fe35—C33	68.88 (14)	C1—C6—H38	119.9 (7)
C30—Fe35—C34	40.89 (14)	C5—C6—H38	120.9 (7)
C31—Fe35—C32	40.91 (16)	C10—C9—H39	119.7 (9)
C31—Fe35—C33	68.85 (15)	C14—C9—H39	120.2 (8)
C31—Fe35—C34	68.77 (14)	C9—C10—H40	119.9 (7)
C32—Fe35—C33	40.90 (14)	C11—C10—H40	120.0 (7)
C32—Fe35—C34	68.81 (14)	C12—C13—H41	119.8 (7)
C33—Fe35—C34	40.92 (14)	C14—C13—H41	120.0 (6)
C8—N16—C19	122.0 (4)	C9—C14—H42	120 (3)
C2—C1—C6	118.8 (4)	C13—C14—H42	120 (3)
C1—C2—C3	120.6 (4)	C19—C20—H43	120.0 (7)
C2—C3—C4	119.4 (4)	C21—C20—H43	119.8 (7)
C2—C3—C8	120.7 (4)	C20—C21—H44	119.9 (9)
C4—C3—C8	119.1 (3)	C22—C21—H44	120.0 (10)
C3—C4—C5	120.2 (3)	C21—C22—H45	119.8 (8)
C3—C4—C7	120.3 (3)	C23—C22—H45	119.8 (8)
C5—C4—C7	119.4 (3)	C22—C23—H46	120.1 (10)
C4—C5—C6	120.7 (4)	C24—C23—H46	119.9 (8)
C4—C5—C17	114.1 (3)	C19—C24—H47	119.8 (8)
C6—C5—C17	122.0 (4)	C23—C24—H47	119.9 (8)
C1—C6—C5	118.8 (4)	Fe35—C25—H48	126.0 (4)
O15—C7—C4	119.8 (4)	C26—C25—H48	126.0 (5)
O15—C7—C12	118.7 (3)	C29—C25—H48	126.1 (5)
C4—C7—C12	118.4 (3)	Fe35—C26—H49	126.0 (4)
N16—C8—C3	121.8 (3)	C25—C26—H49	126.0 (4)
N16—C8—C11	118.7 (3)	C27—C26—H49	125.9 (3)
C3—C8—C11	118.1 (3)	Fe35—C27—H50	126.1 (4)
C10—C9—C14	120.1 (5)	C26—C27—H50	126.1 (3)
C9—C10—C11	118.9 (5)	C28—C27—H50	125.9 (4)
C8—C11—C10	122.0 (4)	Fe35—C29—H51	126.1 (3)
C8—C11—C12	117.5 (3)	C25—C29—H51	126.0 (4)
C10—C11—C12	119.9 (4)	C28—C29—H51	126.0 (4)
C7—C12—C11	119.4 (3)	Fe35—C30—H52	126.0 (4)
C7—C12—C13	119.4 (4)	C31—C30—H52	125.9 (4)
C11—C12—C13	120.0 (4)	C34—C30—H52	126.1 (4)
C12—C13—C14	119.3 (5)	Fe35—C31—H53	126.0 (3)
C9—C14—C13	120.3 (4)	C30—C31—H53	126.0 (5)
C5—C17—C18	158.2 (5)	C32—C31—H53	126.0 (5)
C17—C18—C28	156.8 (4)	Fe35—C32—H54	127.1 (4)
N16—C19—C20	119.0 (4)	C31—C32—H54	126.1 (4)
N16—C19—C24	118.2 (3)	C33—C32—H54	126.0 (4)
C20—C19—C24	119.2 (4)	Fe35—C33—H55	126.1 (4)
C19—C20—C21	119.6 (4)	C32—C33—H55	126.0 (4)
C20—C21—C22	120.0 (5)	C34—C33—H55	126.0 (4)
C21—C22—C23	120.1 (5)	Fe35—C34—H56	125.9 (3)
C22—C23—C24	119.6 (4)	C30—C34—H56	126.0 (4)
C19—C24—C23	120.1 (3)	C33—C34—H56	126.0 (4)
			
C32—Fe35—C28—C27	76.3 (2)	C33—Fe35—C28—C18	1.7 (4)
C33—Fe35—C28—C27	118.3 (2)	C31—Fe35—C33—C32	37.7 (2)
C34—Fe35—C28—C27	160.8 (2)	C34—Fe35—C33—C32	119.3 (3)
C25—Fe35—C28—C29	37.70 (18)	C26—Fe35—C28—C27	−37.65 (18)
C26—Fe35—C28—C29	81.52 (18)	C29—Fe35—C28—C27	−119.2 (3)
C27—Fe35—C28—C29	119.2 (3)	C28—Fe35—C33—C34	118.4 (2)
C30—Fe35—C28—C29	−45.6 (4)	C34—Fe35—C31—C32	−81.7 (2)
C32—Fe35—C28—C29	−164.5 (2)	C34—Fe35—C32—C33	−37.8 (2)
C33—Fe35—C28—C29	−122.6 (2)	C30—Fe35—C33—C32	81.6 (2)
C34—Fe35—C28—C29	−80.0 (2)	C25—Fe35—C32—C31	−46.9 (5)
C25—Fe35—C32—C33	−166.3 (4)	C26—Fe35—C32—C31	−80.0 (2)
C26—Fe35—C32—C33	160.6 (2)	C27—Fe35—C32—C31	−122.2 (2)
C27—Fe35—C32—C33	118.4 (2)	C28—Fe35—C32—C31	−163.74 (19)
C28—Fe35—C32—C33	76.9 (2)	C30—Fe35—C32—C31	37.62 (19)
C30—Fe35—C32—C33	−81.8 (2)	C33—Fe35—C32—C31	119.4 (3)
C26—Fe35—C29—C25	37.65 (17)	C29—Fe35—C33—C32	−163.9 (2)
C27—Fe35—C29—C25	81.50 (19)	C28—Fe35—C34—C30	160.4 (2)
C28—Fe35—C29—C25	119.2 (2)	C29—Fe35—C31—C32	−166.8 (4)
C30—Fe35—C29—C25	−79.7 (2)	C26—Fe35—C33—C34	−165.8 (4)
C31—Fe35—C29—C25	−45.4 (5)	C27—Fe35—C33—C34	160.7 (2)
C33—Fe35—C29—C25	−164.3 (2)	C27—Fe35—C34—C30	−166.1 (4)
C34—Fe35—C29—C25	−122.3 (2)	C33—Fe35—C31—C32	−37.70 (19)
C25—Fe35—C29—C28	−119.2 (2)	C30—Fe35—C31—C32	−119.5 (3)
C26—Fe35—C29—C28	−81.59 (16)	C26—Fe35—C33—C32	−46.5 (5)
C27—Fe35—C29—C28	−37.74 (15)	C27—Fe35—C33—C32	−80.0 (2)
C30—Fe35—C29—C28	161.04 (18)	C28—Fe35—C33—C32	−122.3 (2)
C31—Fe35—C29—C28	−164.7 (4)	C25—Fe35—C34—C30	76.4 (2)
C33—Fe35—C29—C28	76.5 (2)	C19—N16—C8—C3	−24.2 (6)
C34—Fe35—C29—C28	118.43 (18)	C19—N16—C8—C11	169.9 (3)
C25—Fe35—C30—C31	118.1 (2)	C8—N16—C19—C20	−50.6 (6)
C26—Fe35—C30—C31	76.5 (3)	C8—N16—C19—C24	151.0 (5)
C28—Fe35—C30—C31	−166.7 (4)	C6—C1—C2—C3	−11.1 (8)
C29—Fe35—C30—C31	160.2 (2)	C2—C1—C6—C5	13.1 (8)
C32—Fe35—C30—C31	−37.7 (2)	C1—C2—C3—C4	0.3 (7)
C33—Fe35—C30—C31	−81.7 (2)	C1—C2—C3—C8	−169.3 (4)
C34—Fe35—C30—C31	−119.4 (3)	C2—C3—C4—C5	8.3 (5)
C25—Fe35—C30—C34	−122.6 (2)	C8—C3—C4—C7	−5.0 (5)
C26—Fe35—C30—C34	−164.1 (2)	C2—C3—C8—N16	−10.8 (6)
C28—Fe35—C30—C34	−47.4 (5)	C4—C3—C8—N16	179.6 (4)
C29—Fe35—C30—C34	−80.4 (2)	C4—C3—C8—C11	−14.5 (5)
C31—Fe35—C30—C34	119.4 (3)	C2—C3—C8—C11	155.2 (4)
C32—Fe35—C30—C34	81.7 (2)	C8—C3—C4—C5	178.1 (3)
C33—Fe35—C30—C34	37.68 (19)	C2—C3—C4—C7	−174.8 (4)
C31—Fe35—C27—C28	−164.5 (2)	C3—C4—C7—C12	26.6 (5)
C32—Fe35—C27—C28	−122.6 (2)	C5—C4—C7—O15	3.1 (6)
C33—Fe35—C27—C28	−80.1 (2)	C3—C4—C7—O15	−173.8 (4)
C34—Fe35—C27—C28	−46.2 (5)	C7—C4—C5—C17	16.9 (5)
C29—Fe35—C34—C33	−122.4 (2)	C5—C4—C7—C12	−156.5 (3)
C30—Fe35—C34—C33	119.5 (3)	C3—C4—C5—C17	−166.1 (3)
C25—Fe35—C31—C30	−80.2 (2)	C3—C4—C5—C6	−6.2 (5)
C26—Fe35—C31—C30	−122.3 (2)	C7—C4—C5—C6	176.9 (4)
C27—Fe35—C31—C30	−163.7 (2)	C6—C5—C17—C18	56.4 (14)
C29—Fe35—C31—C30	−47.3 (5)	C4—C5—C17—C18	−143.9 (12)
C32—Fe35—C31—C30	119.5 (3)	C4—C5—C6—C1	−4.8 (7)
C33—Fe35—C31—C30	81.8 (2)	C17—C5—C6—C1	153.6 (4)
C34—Fe35—C31—C30	37.7 (2)	O15—C7—C12—C13	4.2 (7)
C25—Fe35—C31—C32	160.3 (2)	C4—C7—C12—C11	−28.5 (5)
C26—Fe35—C31—C32	118.2 (2)	O15—C7—C12—C11	171.7 (4)
C27—Fe35—C31—C32	76.8 (2)	C4—C7—C12—C13	164.0 (4)
C27—Fe35—C25—C26	37.66 (17)	C3—C8—C11—C10	−158.7 (4)
C28—Fe35—C25—C26	81.56 (18)	C3—C8—C11—C12	12.2 (5)
C29—Fe35—C25—C26	119.3 (3)	N16—C8—C11—C10	7.8 (6)
C30—Fe35—C25—C26	−122.3 (2)	N16—C8—C11—C12	178.6 (4)
C31—Fe35—C25—C26	−79.8 (2)	C14—C9—C10—C11	−9.8 (9)
C32—Fe35—C25—C26	−45.8 (4)	C10—C9—C14—C13	5.0 (10)
C34—Fe35—C25—C26	−164.0 (2)	C9—C10—C11—C8	−175.8 (4)
C26—Fe35—C25—C29	−119.3 (3)	C9—C10—C11—C12	13.5 (7)
C27—Fe35—C25—C29	−81.59 (19)	C8—C11—C12—C7	8.7 (5)
C28—Fe35—C25—C29	−37.69 (17)	C10—C11—C12—C13	−12.8 (7)
C30—Fe35—C25—C29	118.5 (2)	C8—C11—C12—C13	176.2 (4)
C31—Fe35—C25—C29	160.9 (2)	C10—C11—C12—C7	179.8 (4)
C32—Fe35—C25—C29	−165.1 (4)	C7—C12—C13—C14	175.2 (6)
C34—Fe35—C25—C29	76.7 (2)	C11—C12—C13—C14	7.8 (8)
C34—Fe35—C32—C31	81.6 (2)	C12—C13—C14—C9	−4.0 (10)
C29—Fe35—C27—C28	37.7 (2)	C5—C17—C18—C28	−173.5 (8)
C32—Fe35—C34—C30	−81.7 (2)	C17—C18—C28—C29	65.2 (11)
C33—Fe35—C34—C30	−119.5 (3)	C17—C18—C28—Fe35	−36.7 (12)
C25—Fe35—C34—C33	−164.1 (2)	C17—C18—C28—C27	−132.3 (9)
C31—Fe35—C32—C33	−119.4 (3)	N16—C19—C20—C21	−171.6 (5)
C27—Fe35—C26—C25	−119.3 (3)	N16—C19—C24—C23	172.9 (5)
C28—Fe35—C26—C25	−81.6 (2)	C24—C19—C20—C21	−13.4 (8)
C29—Fe35—C26—C25	−37.71 (18)	C20—C19—C24—C23	14.6 (8)
C30—Fe35—C26—C25	76.5 (2)	C19—C20—C21—C22	6.1 (10)
C31—Fe35—C26—C25	118.2 (2)	C20—C21—C22—C23	0.3 (11)
C32—Fe35—C26—C25	160.7 (2)	C21—C22—C23—C24	0.8 (12)
C33—Fe35—C26—C25	−165.6 (4)	C22—C23—C24—C19	−8.3 (10)
C25—Fe35—C26—C27	119.3 (3)	C26—C25—C29—Fe35	−59.4 (2)
C28—Fe35—C26—C27	37.69 (18)	Fe35—C25—C29—C28	59.43 (19)
C29—Fe35—C26—C27	81.6 (2)	C29—C25—C26—C27	−0.1 (3)
C30—Fe35—C26—C27	−164.2 (2)	C26—C25—C29—C28	0.1 (3)
C31—Fe35—C26—C27	−122.5 (2)	C29—C25—C26—Fe35	59.3 (2)
C32—Fe35—C26—C27	−80.0 (2)	Fe35—C25—C26—C27	−59.4 (2)
C33—Fe35—C26—C27	−46.3 (5)	C25—C26—C27—C28	0.0 (3)
C29—Fe35—C33—C34	76.8 (2)	C25—C26—C27—Fe35	59.4 (2)
C30—Fe35—C33—C34	−37.66 (19)	Fe35—C26—C27—C28	−59.3 (2)
C31—Fe35—C33—C34	−81.6 (2)	C26—C27—C28—C29	0.0 (3)
C25—Fe35—C27—C26	−37.65 (18)	Fe35—C27—C28—C29	−59.3 (2)
C28—Fe35—C27—C26	−119.3 (3)	Fe35—C27—C28—C18	135.1 (3)
C29—Fe35—C27—C26	−81.6 (2)	C26—C27—C28—Fe35	59.4 (2)
C31—Fe35—C27—C26	76.2 (2)	C26—C27—C28—C18	−165.6 (3)
C32—Fe35—C27—C26	118.1 (2)	C27—C28—C29—Fe35	59.4 (2)
C33—Fe35—C27—C26	160.6 (2)	C27—C28—C29—C25	−0.1 (3)
C34—Fe35—C27—C26	−165.5 (4)	C18—C28—C29—C25	164.6 (3)
C25—Fe35—C27—C28	81.6 (2)	Fe35—C28—C29—C25	−59.42 (18)
C26—Fe35—C27—C28	119.3 (3)	C18—C28—C29—Fe35	−136.0 (3)
C29—Fe35—C34—C30	118.1 (2)	Fe35—C30—C31—C32	59.0 (2)
C31—Fe35—C34—C30	−37.7 (2)	C31—C30—C34—C33	0.2 (3)
C34—Fe35—C28—C18	44.3 (4)	Fe35—C30—C34—C33	−59.1 (2)
C25—Fe35—C28—C27	−81.5 (2)	C31—C30—C34—Fe35	59.2 (2)
C27—Fe35—C34—C33	−46.6 (5)	C34—C30—C31—Fe35	−59.2 (2)
C28—Fe35—C34—C33	−80.1 (2)	C34—C30—C31—C32	−0.1 (3)
C30—Fe35—C28—C27	−164.7 (4)	C30—C31—C32—C33	0.0 (3)
C31—Fe35—C34—C33	81.8 (2)	Fe35—C31—C32—C33	59.1 (2)
C32—Fe35—C34—C33	37.76 (19)	C30—C31—C32—Fe35	−59.1 (2)
C32—Fe35—C33—C34	−119.3 (3)	C31—C32—C33—C34	0.1 (4)
C25—Fe35—C28—C18	162.0 (3)	C31—C32—C33—Fe35	−59.1 (2)
C26—Fe35—C28—C18	−154.2 (3)	Fe35—C32—C33—C34	59.2 (2)
C27—Fe35—C28—C18	−116.6 (4)	C32—C33—C34—C30	−0.2 (4)
C29—Fe35—C28—C18	124.3 (3)	Fe35—C33—C34—C30	59.1 (2)
C30—Fe35—C28—C18	78.7 (6)	C32—C33—C34—Fe35	−59.3 (2)
C32—Fe35—C28—C18	−40.3 (4)		

### Hydrogen-bond geometry (Å, º)

Cg1 is the centroid of the C9–C14 ring.

**Table:**

*D*—H···*A*	*D*—H	H···*A*	*D*···*A*	*D*—H···*A*
C34—H56···*Cg*1^i^	0.87 (1)	2.86 (1)	3.588 (4)	143 (1)

Symmetry code: (i) *x*, *y*−1, *z*.
